# Consequences of COVID-19 crisis for persons with HIV: the impact of social determinants of health

**DOI:** 10.1186/s12889-021-10296-9

**Published:** 2021-02-05

**Authors:** Kristie C. Waterfield, Gulzar H. Shah, Gina D. Etheredge, Osaremhen Ikhile

**Affiliations:** 1 Department of Health Policy and Community Health, Jiann-Ping Hsu College of Public Health, Georgia Southern University, Statesboro, GA USA; 2 Department Chair and Professor of Health Policy and Community Health, Jiann-Ping Hsu College of Public Health, Georgia Southern University, PO Box 8015, Statesboro, GA 30460 USA; 3 FHI 360, Washington, DC, USA

**Keywords:** HIV, COVID-19, Public health, Comorbidities, Consequences

## Abstract

**Background:**

With the indiscriminate spread of COVID-19 globally, many populations are experiencing negative consequences such as job loss, food insecurity, and inability to manage existing medical conditions and maintain preventive measures such as social distancing and personal preventative equipment. Some of the most disadvantaged in the COVID-19 era are people living with HIV/AIDS and other autoimmune diseases.

**Discussion:**

As the number of new HIV infections decrease globally, many subpopulations remain at high risk of infection due to lack of or limited access to prevention services, as well as clinical care and treatment. For persons living with HIV or at higher risk of contracting HIV, including persons who inject drugs or men that have sex with men, the risk of COVID-19 infection increases if they have certain comorbidities, are older than 60 years of age, and are homeless, orphaned, or vulnerable children. The risk of COVID-19 is also more significant for those that live in Low- and Middle-Income Countries, rural, and/or poverty-stricken areas. An additional concern for those living the HIV is the double stigma that may arise if they also test positive for COVID-19. As public health and health care workers try to tackle the needs of the populations that they serve, they are beginning to realize the need for a change in the infrastructure that will include more efficient partnerships between public health, health care, and HIV programs.

**Conclusion:**

Persons living with HIV that also have other underlying comorbidities are a great disadvantage from the negative consequences of COVID-19. For those that may test positive for both HIV and COVID-19, the increased psychosocial burdens stemming from stress and isolation, as well as, experiencing additional barriers that inhibit access to care, may cause them to become more disenfranchised. Thus, it becomes very important during the current pandemic for these challenges and barriers to be addressed so that these persons living with HIV can maintain continuity of care, as well as, their social and mental support systems.

## Background

The transmission of the severe acute respiratory syndrome coronavirus 2 (SARS-CoV-2), a novel virus that causes COVID-19 infection, seems to be indiscriminate. The negative consequences of COVID-19 for some populations are more severe than others, including job loss, food insecurity, inability to manage existing conditions, and inability to maintain preventive measures such as social distancing and use of personal protective equipment (PPE). Those who live in poverty have less control of their living arrangements and their immediate environment, thus the barriers that they are facing when trying to protect themselves and their families are greater than those that are not living in poverty [1, 2]. Among the most disenfranchised and most disadvantaged in the COVID-19 era are people living with HIV/AIDS, people at risk of contracting HIV such as sex workers, people who inject drugs, and men who have sex with men, and people with other autoimmune diseases [3]. As the pandemic has led to significant changes in health service delivery and amplified fears of increased death and illness, the health inequities and the consequences of these changes among various subgroups of those living in HIV/AIDS, or at risk of contracting HIV must be reviewed through the lens of social determinants of health (SDoH).

## Main text

### Cross-cutting circles of disadvantages and health inequity for PLHIV

As the number of new HIV infections decrease globally, there are still subpopulations that remain at higher risk of infection and have limited or no access to prevention, care, and treatment. The populations that are the hardest hit are those that also lack power and experience high levels of food insecurity, poverty, drug abuse, incarceration among men, and those suffering from other infectious and chronic diseases [4, 5].

### Disadvantages for PLHIV with other underlying comorbidities

For persons living with HIV (PLHIV) and persons at risk for contracting HIV, many SDoH will change for the worse during the COVID-19. The PLHIV, especially people of color and with additional underlying conditions, have an elevated risk of SARS-CoV-2 infection. There is emerging empirical evidence that the risk of SARS-CoV-2 transmission and COVID-19 deaths is higher for immunocompromised persons, people of color (especially those of African descent), or those with certain pre-existing conditions such as cancer, hypertension, heart disease, lung disease, and diabetes [6]. According to the Centers for Disease Control and Prevention (CDC), 16.7% of COVID-19 patients in intensive care are immunocompromised with underlying health conditions [4] and their chances of recovery are much lower. People of color, especially those of African descent, and those living in resource-poor countries are significantly more likely to experience access to care barriers and mortality related to COVID-19 [1, 2]. PLHIV with other underlying comorbidities (e.g., diabetes, cardiovascular disease, lung disease) are at an even greater disadvantage than PLHIV without additional underlying comorbidities [6]. Before the advent of effective combination antiretroviral therapy (ART), advanced HIV infection was a risk factor for complications of other respiratory infections, and severe respiratory distress [7]. COVID-19 increases the likelihood of a syndemic effect on PLHIV with underlying comorbidities. For example, research has shown that HIV/TB co-infection not only augments the immunopathology of HIV and accelerates the damaging progression of the disease; but that ART initiation must be timed in order to increase adherence and decrease adverse effects of drug interactions [8] especially for those utilizing HIV clinics in Low- and Middle-Income Countries (LMIC).

The social support and community mobilization approaches for the management of HIV/AIDS, as opposed to individual-level approaches alone, have been shown to improve antiviral medication adherence [9]. However, due to the enforcement of social distancing and other restrictions in the current pandemic, there has been a shift in the mechanism by which PLHIV receive such social support. A breakdown in social support would likely impact medication adherence as individuals with a low CD4 cell count and PLHIV not on ART are at a higher risk of becoming extremely sick with COVID-19 infection [6]. It is imperative to explore and monitor the adequacy of the current guidelines for the management of PLHIV with other underlying comorbidities. However, ensuring PLHIV have access to their antiviral refills, as well as the maintenance of their social support including material and mental support from community, family, and friends [9] may pose a challenge; hence, the need for the establishment of proper linkage to care. Additionally, PLHIV in LMIC and rural areas may experience additional barriers that inhibit access to care such as the lack of adequate technology for telemedicine, lack of transportation to open clinics, and additional costs and complexity required to provide care during the current pandemic.

### Double-jeopardy for COVID-HIV social stigma

Shiau and colleagues (2020) note that PLHIV not only faces a higher risk of severe illness from COVID-19 infection due to compromised immune systems, they also have increased psychosocial burdens emanating from stress and isolation [10]. The compounded stigma associated with HIV-positive status and SARS-CoV-2 may lead to an array of mental health issues comprising psychological disorders such as stress, depression, sleep disorder, substance abuse, anxiety, schizophrenia, and personality disorders, which may trigger somatoform disorders. Instances of blatant wrongful attributions of certain race categories as a source of SARS-CoV-2 spread inflicts additional harm [11]. The compounded HIV/COVID-19 stigma may lead to failure to seek care. The suffering and social stigma are worse for disenfranchised and low socioeconomic status (SES) individuals and subgroups, creating health disparities. COVID-19-related cancellations of patient procedures and the non-availability of doctors will make the systems too overburdened to care for these groups [12]. The suffering and social stigma are worse for disenfranchised and low SES individuals and subgroups, creating health inequities and disparities. A recent study suggests that the intersection of social stigma and other social and economic disadvantages among marginalized people, particularly in under-resourced communities, face health disparities in COVID-19 prevention and treatment. Those health disparities stem from a lack of access to care and the inability to practice recommended preventive practices as proper social distancing [13]. An inclination to escape social stigma by suppressing information about SARS-CoV-2 positive persons’ contacts with others is a significant barrier to preventive services such as contact tracing and recommended regular reporting of compliance concerning compliance among persons in quarantine.

### Elevated COVID-19 risk for older adults with HIV or at risk for HIV

According to the CDC and World Health Organization, the prevalence rate among confirmed COVID-19 cases in older adults is approximately 31%, and the death rates from COVID-19 increase with age (60–69 years old 3.6%, 70–79 years old 8.0%, and 80+ years old 18.8%) [14]. Since the identification of SARS-CoV-2 in December 2019, the most common comorbidities among COVID-19 patients with the highest mortality rates (6.0 to 10.5%) and worst health outcomes are cardiovascular disease, chronic respiratory disease, obesity, and diabetes [15]. Even though older adults living with HIV or at risk for HIV make up a small portion of the overall global population, they represent the segment of the population that is at greater risk of having these comorbidities due to changes in their bodies as they get older as well as the effects of an unhealthy lifestyle and/or heredity. So, while the overall projected case fatality rate (CFR) for all confirmed cases has decreased since February 2020, the CFR for older adults has stayed relatively the same and for those over the age of 80 has increased [16].

### COVID-19 risk for orphans and vulnerable children in LMIC

Individuals dependent on other people for care and support, and individuals without self-autonomy, are among the most vulnerable persons around the world. Orphans and Vulnerable Children (OVC) in Low- and Middle-Income Countries comprise a subset of these, with the added burden of having lost one or both parents to HIV and being infected or affected by HIV (USG PEPFAR definition). If OVC cannot live with their biological parents, they often live with extended family members, in families that experience greater physical/economic/psychosocial hardships than others, on the street, or in group homes.

Even before the COVID-19 pandemic, OVC in LMIC were known to perform less well in school and be more likely to lag in grade-for-age, suffer from more abuse, experience higher rates of unwanted pregnancies, and have less medical attention than non-OVC. OVC depend on their caregivers, some of whom have HIV themselves. In many countries, HIV programs have been sharply affected, with care and treatment clinic hours being reduced, medical personnel re-deployed from HIV services to COVID-19 services, community health workers’ movement restricted such that they are unable to do mobile outreach or home visits, supply chains disrupted, etc. In the medical field and other associated sectors, these structural changes, coupled with population movement restrictions or total lockdowns, food, and other commodities’ scarcities, unemployment, and social unrest, contribute to a hotbed of tension, anxiety, and sometimes, most unfortunately, desperation.

Incidences of interpersonal violence (IPV) are on the rise. IPV victims are usually those without power; children are likely victims. Without the means to fight back, including the emotional or cognitive maturity to understand their rights, they too often fall into a cycle of abuse. OVC is dependent on others. They need the structure of a caring and enabling environment, and if they have HIV, enrollment in treatment, and access to antiretroviral therapy and viral load testing. Peer support groups for HIV-positive individuals have been demonstrated to increase coping mechanisms and increase adherence. Support groups, where population movement has been restructured, are no longer feasible. OVC may suffer the most because of the doubly fragile lives -- being a single or double-orphan, and infected or affected by HIV. When the lives of a population are severely disrupted and threatened, the most vulnerable suffer the worst.

### Homelessness and unemployment among PLHIV and those at risk for HIV

Considerable work has been done to show the impact of homelessness on health and wellbeing. Many homeless individuals also suffer from substance abuse, which complicates their mental status and constitutes a risky behavior for the spread of infectious disease. Homeless shelters have been shown to increase the spread of infectious diseases such as SARS and HIV [17], thus, homeless individuals with HIV and homeless individuals that are at risk of contracting HIV (i.e. intravenous drug users, sex workers, and men that have sex with men) are at a higher risk of being infected with COVID-19 [3, 17]. The synergistic effect of these poor social conditions further exacerbates the need to act, because homeless shelters are not health centers and many of them tend to lack the necessary resources needed to curtail the spread of infectious diseases among those staying within the shelters. When seeking healthcare services, those with no access to outpatient care including homeless individuals, migrants and asylum seekers living with HIV often resort to the emergency room; however, since the beginning of the COVID-19 pandemic, many emergency departments have had to change their procedures in order to properly manage the influx of COVID-19 patients and at times have had to restrict access to non-COVID-19 patients (which included PLHIV and those at risk for HIV). These changes in procedures have in turn caused a reduction in access to care for all homeless individuals, especially PLHIV that are in need of ART. Currently, there are projections that the number of homeless individuals may rise due to the surge in the rate of unemployment that is related to the closure of businesses during the COVID-19 lockdowns.

In order to curtail future transmission of COVID-19, health and city officials need to consider the impact of homelessness and unemployment as possible drivers of the spread of infection. Effective communication, financial resources, food insecurity, management of chronic diseases, respite care, personal protection, and housing [9] are issues that should be addressed among this vulnerable population.

### Other subgroups of PLHIV

Other subgroups of PLHIV, and those at risk for contracting HIV, that seem to be disproportionately affected by the current pandemic live in LMIC, rural areas, and high poverty/low-income areas. COVID-19 has also been shown to affect persons of different races and ethnicities differently due to their risk factors for certain chronic diseases such as cardiovascular disease, chronic respiratory disease, obesity, and diabetes.

Before the current pandemic situation, many LMIC, rural, and poverty-stricken area residents experienced limited or no access to health care. The problem has gotten exponentially worse due to the decrease in practitioners’ availability and increased demand for services for active COVID-19 cases. Many clinics and hospitals have had to change how they process and manage patients, which has in turn, caused either a cancellation or delay in treatments for non-COVID-19 patients (including PLHIV). These COVID-19-related cancellations of patient procedures and non-availability of doctors impact clinics and hospitals to the extent that many are threatened with closures, ultimately imposing unemployment and financial hardships for LMIC, rural, and high-poverty/low-income communities. For those patients that are given the option of utilizing telemedicine and telehealth, many of them lack the necessary technology in order to participate in these types of appointments. Thus, for many PLHIV, their ability to maintain their ART regiment is proving to be an increasing challenge.

Additionally, many residents of LMIC, rural, and poverty-stricken areas have low literacy levels, due to their socioeconomic and educational status. A person’s literacy level impacts their health literacy levels and their overall health outcomes. Studies show that a person’s literacy level has a direct effect on their ability to understand health information, their knowledge of health service availability, their decision-making capability, their anxiety level during physician consultation, and their adherence to medications and other treatment protocols [18]. In order for public health and healthcare to implement successful responses to infectious disease outbreaks, the population needs to be able to understand the information that is being presented to them, follow health instructions and guidance, and ultimately adopt the prevention behaviors recommended. This becomes especially important for PLHIV, people at risk for HIV, sex workers, people who inject drugs and people with other autoimmune diseases. Thus, COVID-19 has brought the issue of low health literacy to the forefront, especially during the efforts to ensure that the information can be understood by all and stop the spread of the disease.

One of the most definitive statements that has been made during the current pandemic is that SARS-CoV-2 does not discriminate. Unfortunately, a sobering reality is that global trends are showing that certain racial and ethnic groups are at higher risk of developing more severe cases of COVID-19. These populations included those racial and ethnic groups that are at higher risk for chronic illnesses such as high blood pressure, lung disease, kidney disease, and diabetes. When these populations reside in LMIC, rural, and poverty-stricken areas or have low health literacy rates, their risk of being severely affected by COVID-19 increases even more.

### A call for the healthcare sector to work with HIV programs

Although it is reasonable to have repurposed health care personnel from the HIV arena to the COVID-19 arena, we will need these professionals, or new ones, back into HIV. COVID-19 has taken a dramatic toll on HIV programs. Lockdowns and associated changes have hampered the ability to test new PHIV, diagnose positives, get newly diagnosed positive persons on treatment, continue adherence to treatment (including dispensing of antiretroviral drugs (ARVs)), and ultimately lower rates of viral suppression. Those associated changes include closing clinics, community health workers’ inability to visit PLHIV, the inability of support groups to meet, and the shortage of personal protective equipment for health providers. Evidence already shows an overall decline in persons coming to facilities for HIV testing, as well as, clinical care and treatment. Mitigation measures are in place, including self-testing, online appointment scheduling, internet or mobile phone-based (e.g. WhatsApp) support groups, virtual counseling, fast-tracked ARV multi-month dispensing if it was not already the national policy, differentiated drug delivery including home delivery, etc. Nonetheless, these measures may not be sustainable and may not be enough to close the gap in care and treatment. Health care workers, both facility- and community-based, will be needed now more than ever in HIV programs.

HIV programs can learn from other sectors, and communication and sharing of lessons learned across sectors will be critical. We cannot return to our silos. Different sectors faced some similar challenges, but also some unique to their field. Lessons to be learned, among others, include the best ways to address supply chain barriers, social behavior communication change, fear-mongering and real-time rumors in the digital age, task shifting, professionalization of para-professionals (i.e., the elevation of the cadre of workers with fewer skills regarded in a more positive light, now that we realize they are essential).

COVID-19 may have crowded out other services. Not only were health care workers shifted to work on COVID-19 which reduced their availability to provide routine and even emergency care, but patients themselves put off visits to clinics or hospitals. Patients arriving at health care institutions were often sicker than they would have been in the past due to delays (self-imposed or not) in seeking care. Careful routine data should be examined across sectors and programs to examine this possible crowding-out effect. Data-based evidence will prepare us to tackle sector needs during future public health emergencies so that we do not have a secondary crisis once people return for care.

### Need for partnerships and synergies between public health program, healthcare and HIV programs

Public and non-profit agencies’ infrastructure for the prevention and treatment of HIV around the globe has matured to meet the challenges presented by HIV. Organizations constituting this infrastructure around the globe are experienced in community partnerships, screening and contact tracing for HIV. Resource stewardship, staff training, and leadership capacity building for these organizations may present a cost-effective opportunity for resource-poor countries to address COVID-19 prevention and management needs for PLHIV. This existing infrastructure can also be strengthened and expanded to assist with the COVID-19 crisis among HIV-negative individuals. For instance, existing labs for HIV testing may also be easily supported to expand their services for SARS-CoV-2 testing. The contact tracing staff may be easily trained to expand their contact tracing for SARS-CoV-2 infection and train the new staff, using their experience and best practices in confidentiality, communications, community engagement, and use of technology. Additionally, public health partnerships with community-based organizations can reduce the burden of low or no access to health care services during the current pandemic when many health care providers are closed or providing few services. This model of use of existing HIV prevention infrastructure to assist with COVID-19 efforts may also be useful due to its cost-effectiveness and efficiency in developed countries. For instance, in the United States, the CDC encourages HIV service providers to be innovative in prioritizing COVID-19 services while still focusing their efforts on HIV prevention and treatment [6].

Given the stigma and stress associated with HIV-positive status, any well-coordinated efforts to combat COVID-19 will require partnerships and synergies between public health programs, healthcare providers, and HIV programs for proper screening, and serology testing of asymptomatic PLHIV, as well as management of persons with COVID-19 and HIV-positive statuses. These partnerships may need to persist even when COVID crisis is over, in order to study the relative vulnerability of PLHIV to COVID-19. Serology testing is possible, even months or years after infection, to detect whether the asymptomatic persons were actually infected.

Fear of the unknown, lack of specific information about symptoms, and prevention strategies are known to mislead the masses, resulting in challenges for programs attempting to tackle the COVID-19. Gallup surveys in 20 countries worldwide have revealed that lack of awareness and knowledge among the masses leads people to believe that the COVID-19 threat is not real. People quote anecdotal evidence and social media discussions, such as not having seen a real COVID victim. The awareness-raising can help such people realize that Coronavirus cannot be seen, that there are a large number of asymptomatic patients, and those with severe illness may have been isolated and out of sight of people. Arranging for PPEs for care providers is necessary for effectiveness in contact tracing, screening, testing, and care related to COVID-19. Even when knowledge about SARS-CoV-2 exists, many underprivileged groups may not comply with recommended prevention procedures due to crowded spaces, jobs that make social distancing impossible, and lack of PPEs. Coordinated efforts from community stakeholders, civil society, public health agencies, and healthcare providers may be essential for addressing health inequities resulting from social stigma and poor access to PPE and preventive services such as screening and contact tracing [13].

## Conclusions

PLHIV with other underlying comorbidities (e.g., diabetes, cardiovascular disease, lung disease) are at an even greater disadvantage and have an elevated risk of COVID-19 infection. The negative consequences of COVID-19 for PHIV can be more severe than for those without HIV. The negative consequences for PHIV can be more severe if they are elderly, OVC, or residents of LMIC, rural, and poverty-stricken areas. Examples of negative consequences include unemployment, food insecurity, and inability to manage pre-existing conditions and preventive measures such as social distancing and PPE use. The compounded stigma associated with HIV-positive status and SARS-CoV-2 may increase psychosocial burdens emanating from stress and isolation for the disenfranchised and low SES individuals. Additionally, PLHIV that reside in LMIC and rural areas may experience additional barriers that inhibit access to care, such as the lack of adequate technology, lack of transportation, and additional costs of care.

Globally, public and non-profit health agencies’ infrastructure has matured to meet the challenges presented by HIV and are experienced in community partnerships, screening, and contact tracing. Many of these agencies have done considerable work to show the impact on the health and wellbeing of homeless PLHIV. Unfortunately, many of these homeless individuals also suffer from substance abuse, which can hinder their mental status and increase the likelihood of engaging in risky behavior. Additionally, homeless shelters have been shown to increase the spread of infectious diseases, which places homeless PLHIV at an even greater risk of contracting COVID-19.

During the current pandemic, PLHIV that utilize health care institutions were often sicker than they would have been in the past due to delays (self-imposed or not) in seeking care. Thus, coordinated efforts from community stakeholders, civil society, public health agencies, and healthcare providers may be essential for addressing health inequities for PLHIV resulting from social stigma and poor access to PPE, preventative services and treatment. The most important challenges for PLHIV that need to be addressed during the current pandemic are access to their antiviral refills and maintenance of their social and mental systems; henceforward, the necessity for the establishment of proper linkage to care and care providers.

## Data Availability

Not applicable.

## Abbreviations

ART: Antiretroviral therapy
ARVs: Antiretroviral drugs
CFR: Case fatality rate
CDC: Centers for Disease Control and Prevention
IPV: Interpersonal violence
LMIC: Low- and Middle-Income Countries
SES: Socioeconomic status
MTb: *Mycobacterium tuberculosis*
OVC: Orphans and Vulnerable Children
PPE: Personal protective equipment
PLHIV: Persons living with HIV
SARS-CoV-2: Severe acute respiratory syndrome coronavirus 2
SDoH: Social determinants of health
